# Supplementary material for: Determining the current size and state of subvolcanic magma reservoirs
Source: Nat Commun. 2020 Nov 5;11:5477. doi: 10.1038/s41467-020-19084-2 (PMC7644707; doi:10.1038/s41467-020-19084-2)
Supplement: Supplementary file 1 — Supplementary Information [file 41467_2020_19084_MOESM1_ESM.pdf]

Supplementary Information for

Estimating the current size and state of subvolcanic magma reservoirs

Gregor Weber[1], Luca Caricchi[1], José L. Arce[2], and Axel K. Schmitt[3]

[1] Department of Earth Sciences, University of Geneva, Geneva, Switzerland.

[2] Instituto de Geología, Universidad Nacional Autónoma de México, Coyoacán, México.

[3] Institut für Geowissenschaften, Universität Heidelberg, Heidelberg, Deutschland.

Corresponding author: Gregor Weber (gregor.weber@unige.ch)

Supplementary Tables

Supplementary Table 1: Sampling locations and description of studied samples.

Sample Number	Eruption	Latitude	Longitude	Altitude (m)	Notes
GW-18-07	Upper Toluca Pumice (UTP)	19.135076	-99.667614	3014	Zaragoza section, Sampling of juvenile vesicular pumices in 3 stratigraphic levels
GW-18-11	Middle Toluca Pumice (MTP)	19.082191	-99.656214	2914	MTP sample in river section sampled in 3 stratigraphic levels as juvenile pumices
GW-18-02	Block and Ash Flow 28 (BAF)	19.183663	-99.664581	2870	Juvenile clasts in Block and Ash Flow below Lower Toluca Pumice in LTP Zacango quarry
GW-16-20-22	White Quarry Flow (WQ)	19.230935	-99.782203	2950	Quarry on road 134 near Zinacantepec, massive pyroclastic flow sequence, pumice sampled in 3 stratigraphic levels

Supplementary Figures

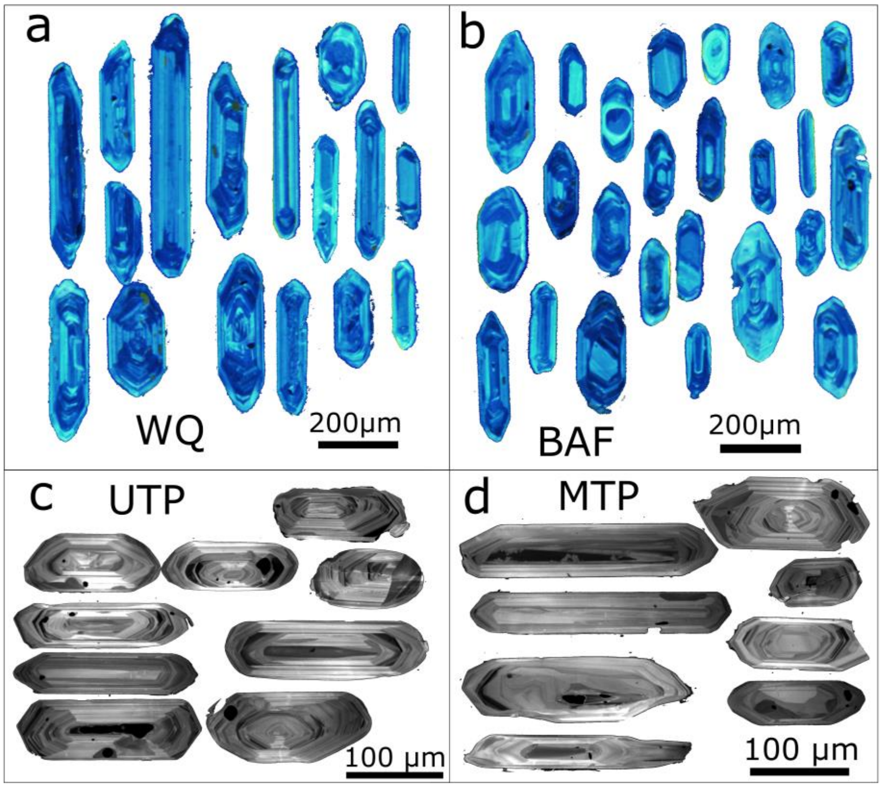

Supplementary Figure 1: Cathodoluminescence (CL) textures of zircon crystals from Nevado de Toluca volcano. a) Optical CL textures of zircons from the WQ eruption. b) Optical CL zonation textures from BAF eruption sample. c) Upper Toluca Pumice (UTP) CL zircon zoning textures obtained by SEM imaging. d) Middle Toluca pumice (MTP) CL zircon textures.

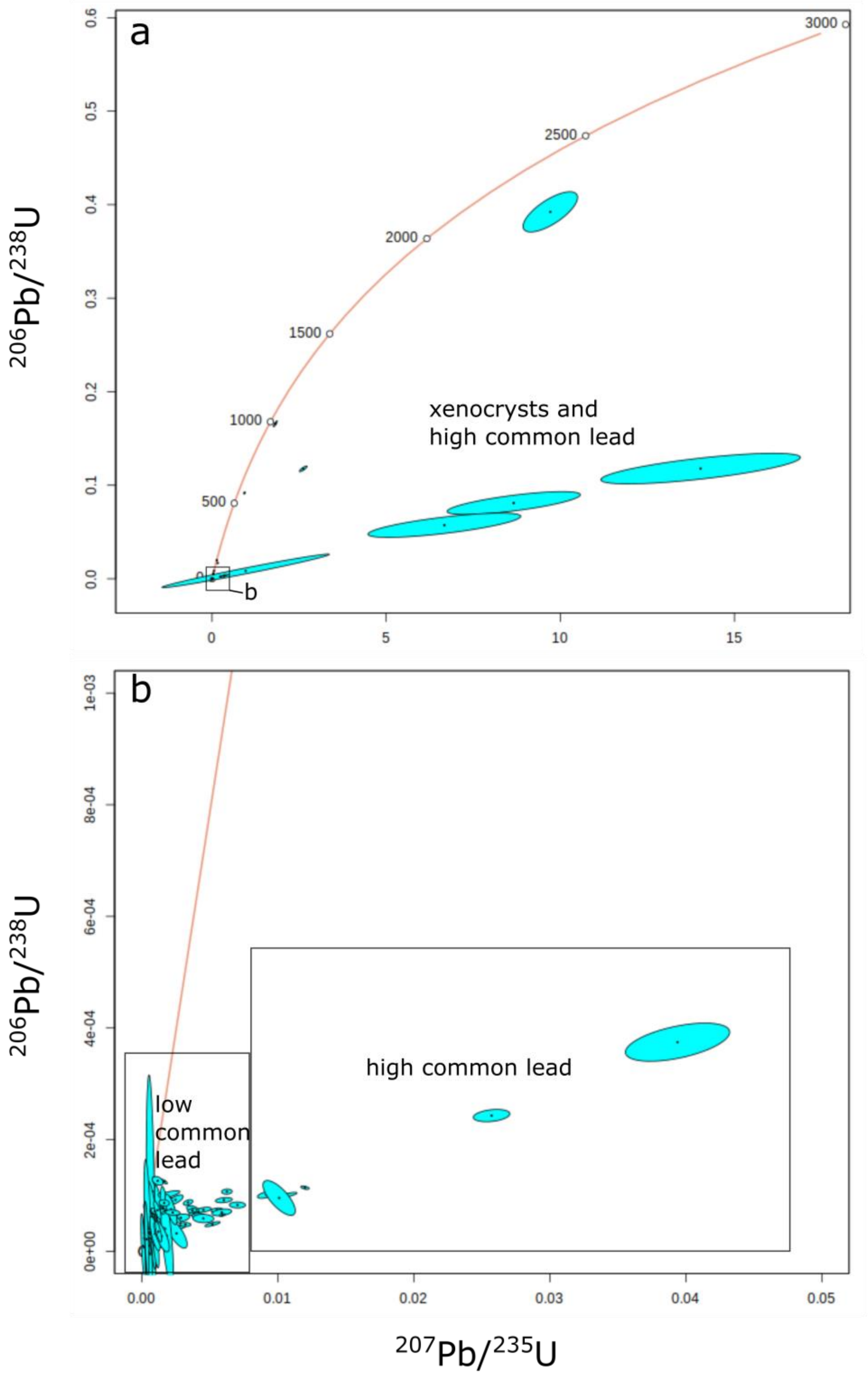

Supplementary Figure 2: $^{206}Pb/^{238}U$ versus $^{207}Pb/^{235}U$ Concordia diagrams for Nevado de Toluca zircon analyzed by LA-ICP-MS. a) Discordant and concordant ages for xenocrystic zircons. b) Young zircons that showed potentially high common lead and discordant ages were excluded from the analysis.

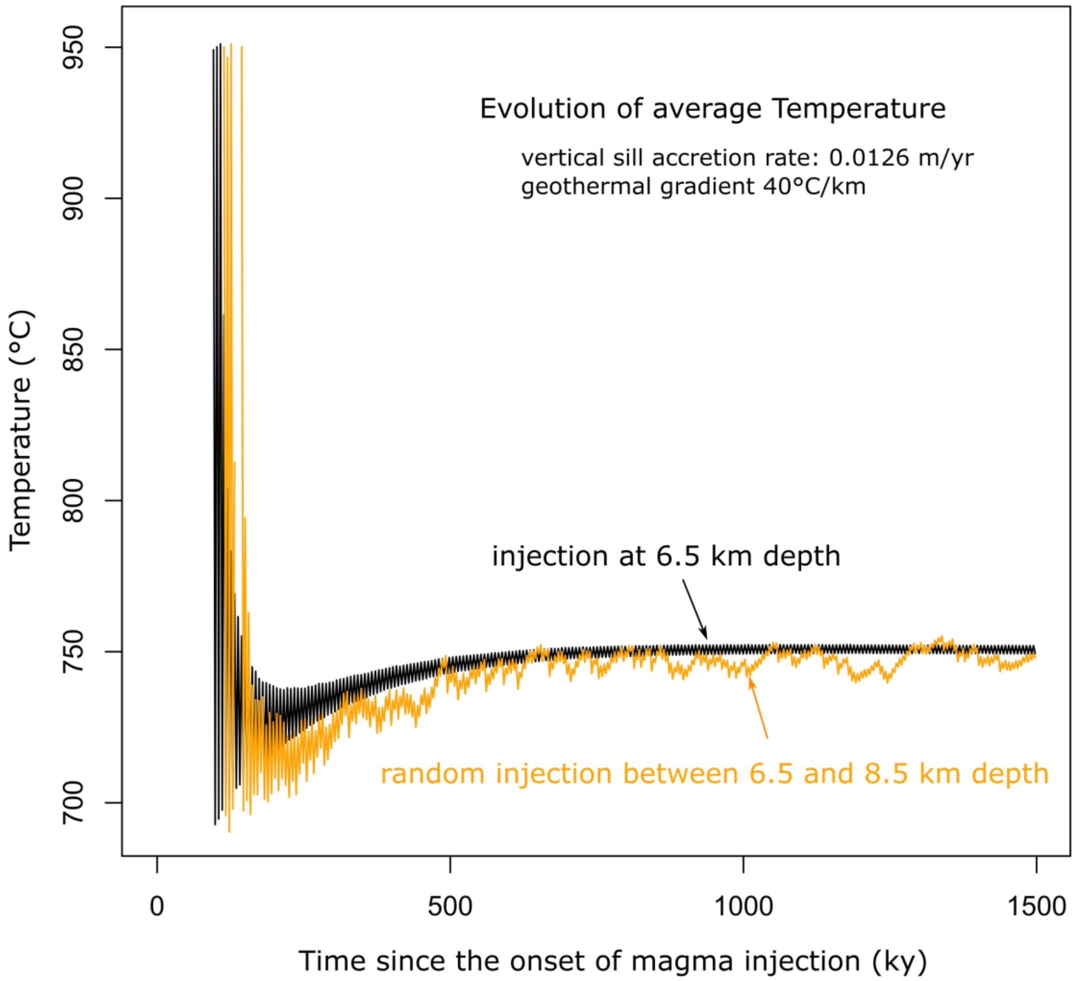

Supplementary Figure 3: Evolution of average intrusion temperature for magma reservoirs built with vertical sill accretion rate of 0.0126 m/yr and initial geothermal gradient of 40°C/km. Black curve indicates temperature evolution for a reservoir built by stationary injection at 6.5 km depth. Orange curve demonstrates temperature evolution of an intrusion built by random injection between 6.5 and 8.5 km depth. Note that the overall temperature-time trends for the 2 curves are similar.

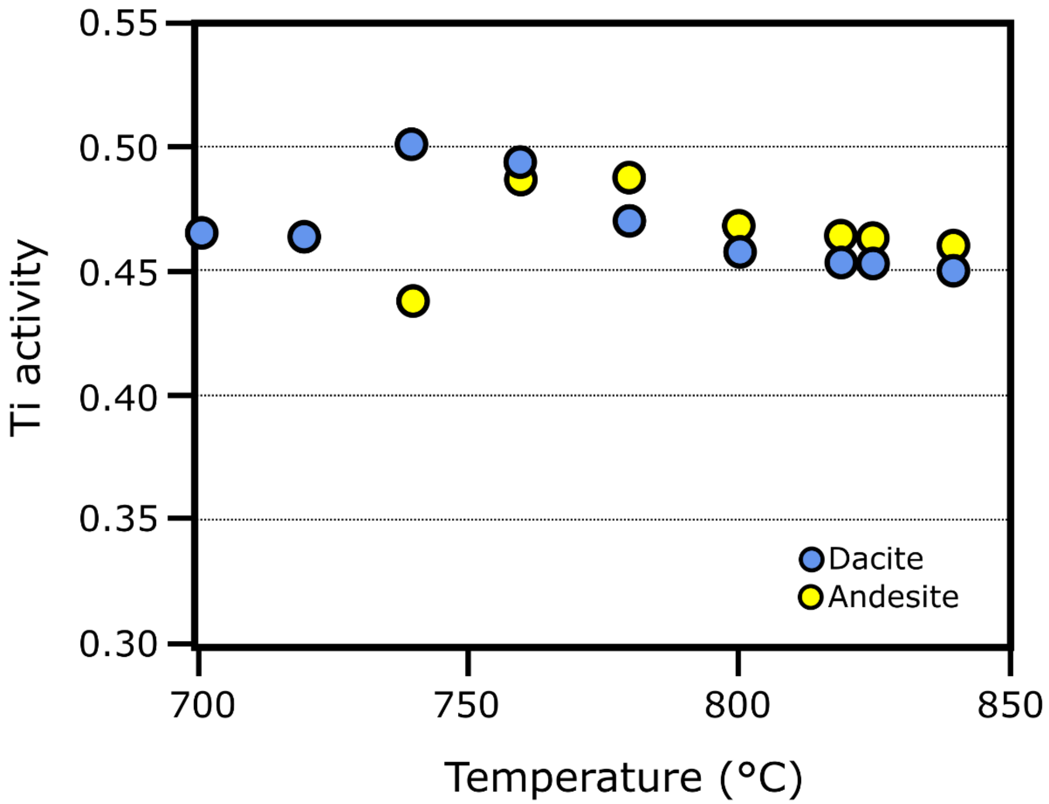

Supplementary Figure 4: Ti activity as a function of temperature for a typical andesite (yellow dots) and dacite (blue dots) from Nevado de Toluca volcano. The Ti activity was calculated from the affinity of rutile for different temperatures using rhyolite-MELTS (Gualda et al., 2012). Oxygen fugacity in the was buffered at NNO in the calculations. Compositions used in the MELTS-simulations were taken from Weber et al., (2019) (samples: GW-16-04 and GW-16-03) with 5 to 6 wt. % added H_2O and absent CO_2.

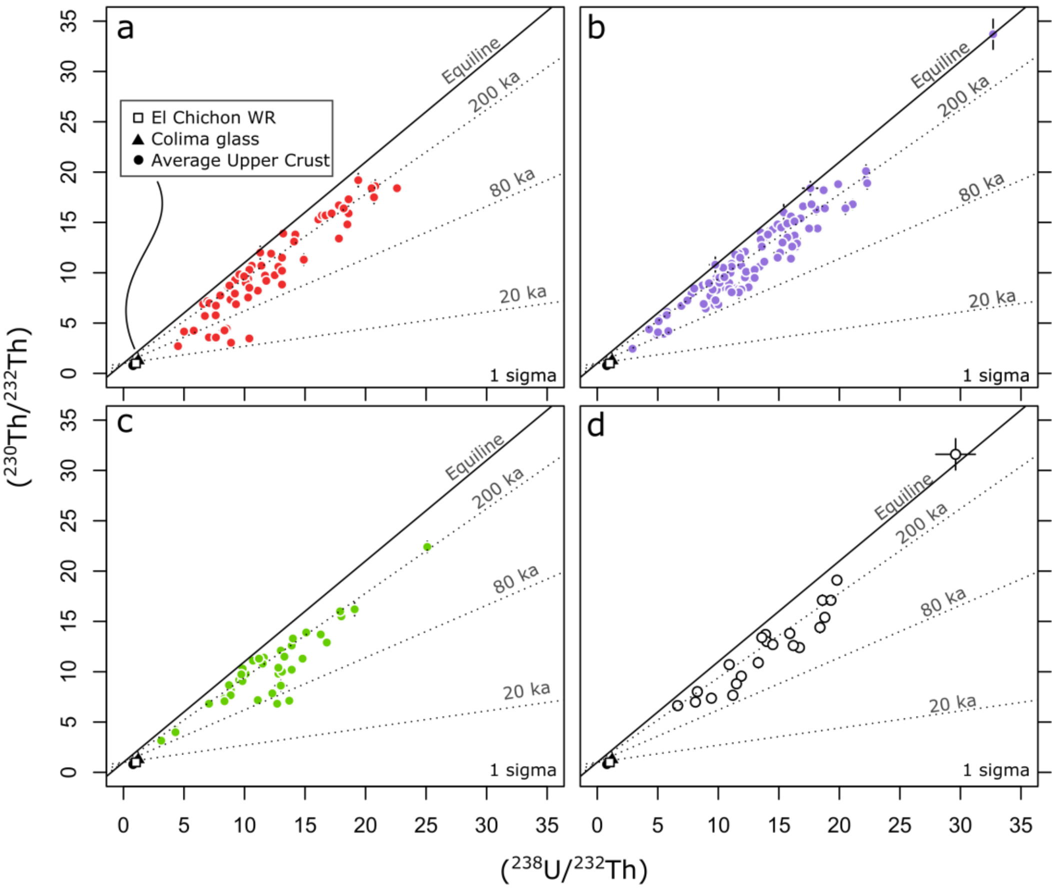

Supplementary Figure 5: (^{230}Th/^{232}Th) versus (^{238}U/^{232}Th) isochrone diagrams for zircons from Nevado de Toluca volcano. Whole rock compositions from El Chichon volcano (open square) (Pickett and Murrell, 1997), which were used to calculate 2-point isochrones ages, as well as average Upper Continental Crust (black dot) (Rudnick and Fountain, 1995) and glass from Colima volcano (black triangle) (Reubi et al., 2017) are shown. a) Upper Toluca Pumice (UTP). b) Middle Toluca Pumice (MTP). c) Block and Ash Flow 28 (BAF). d) White Quarry Flow (WQ). 1sigma error bars are shown.

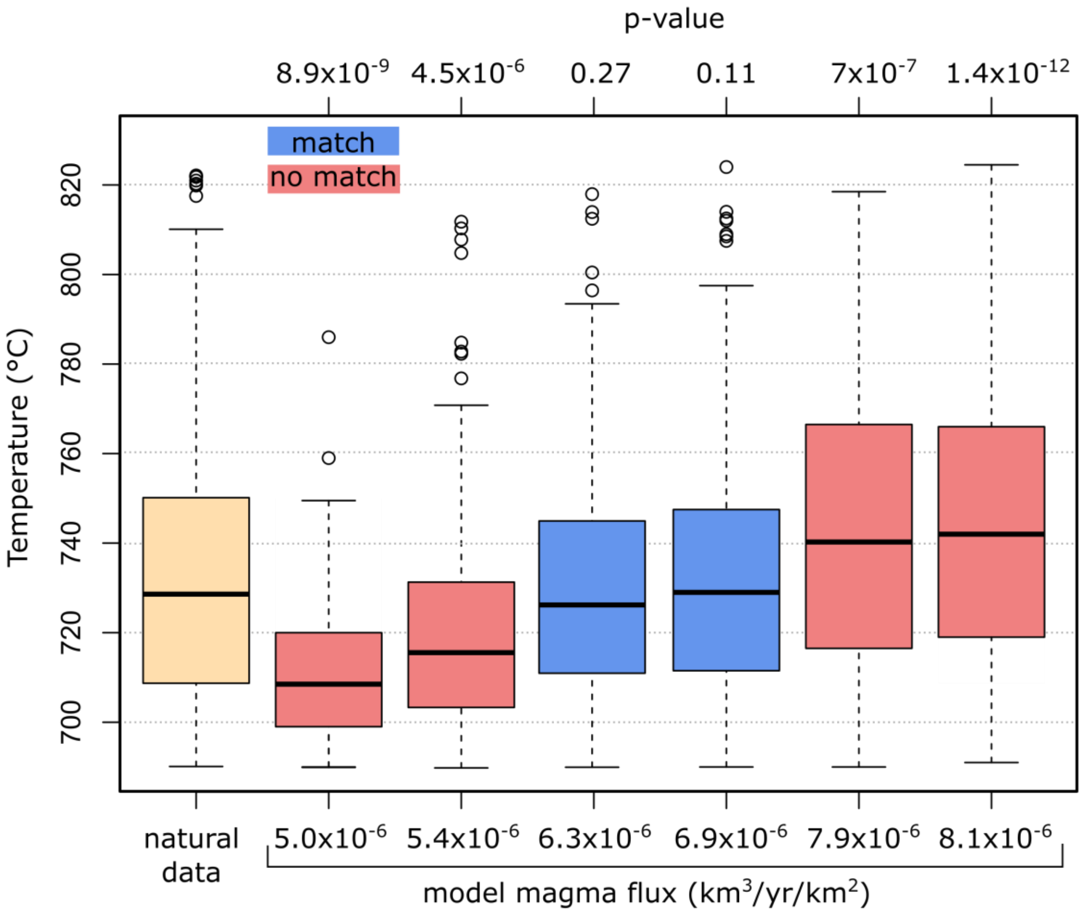

Supplementary Figure 6: Temperature distributions of natural data (tan) and numerical models run with different magma fluxes. A 2-sample Welch's t-test was used to compare the means of natural and modelled temperature distributions. P-values of this comparison are indicated on top of the diagram. Matching temperature distributions between model and natural data are shown by blue boxplots. Red boxplots indicate no match of temperature distributions.

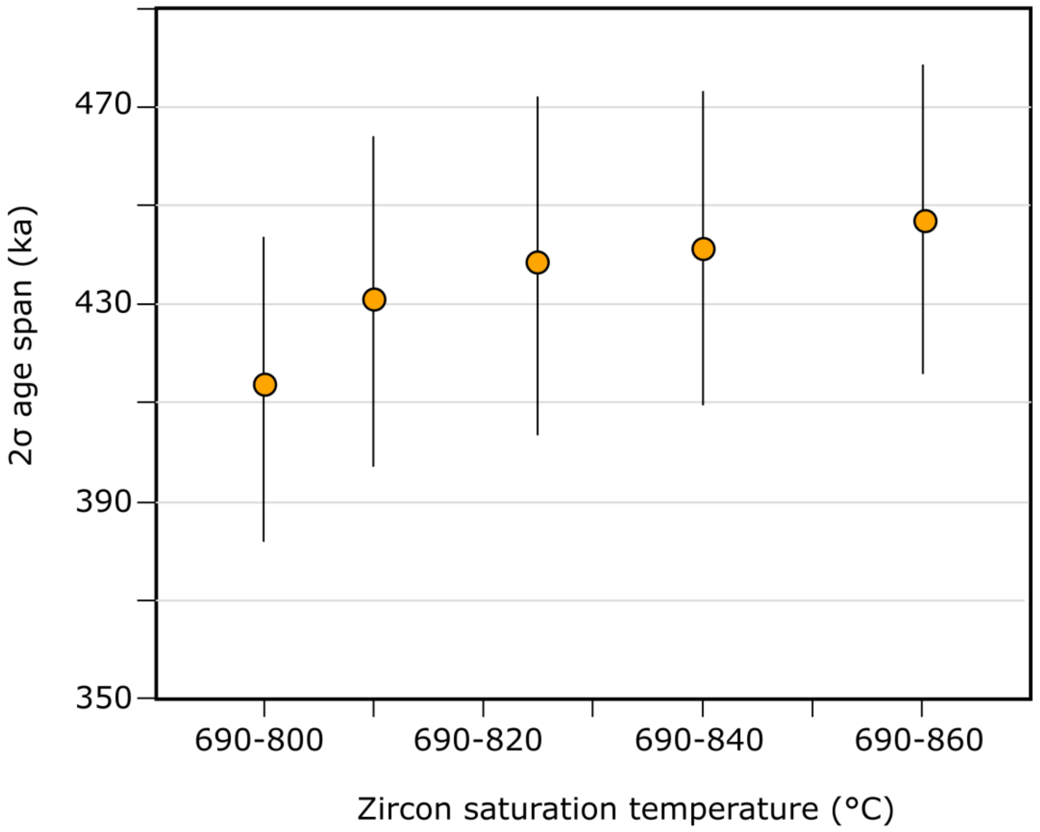

Supplementary Figure 7: Impact of changing zircon saturation temperature range (°C) on the 2σ age spread of synthetic zircon age distributions. All calculations were performed using a magma flux of 0.0091 $km^3/km^2/yr$, a 1.25 Ma magma injection episode and magmatic system radius of 10 km. The error bars indicate the 95% confidence interval of the synthetic 2σ zircon age spans.
