# Supplementary material for: Determining the current size and state of subvolcanic magma reservoirs
Source: Nat Commun. 2020 Nov 5;11:5477. doi: 10.1038/s41467-020-19084-2 (PMC7644707; doi:10.1038/s41467-020-19084-2)
Supplement: Supplementary file 3 — Description of Additional Supplementary Files [file 41467_2020_19084_MOESM3_ESM.pdf]

Description of Additional Supplementary Files

File name: Supplementary Dataset 1
Description: The accompanying R code (created in RStudio version 3.6.3) computes synthetic zircon age populations from the thermal output and calculates the 2σ zircon age spans with 95% confidence interval. The zircon saturation range and the number of synthetic zircon analyses can be specified in the code."

File name: Supplementary Dataset 2
Description: Thermal model outputs (T-t histories). Each column records the T evolution of a particular position in the magma reservoir. The first column shows the time progression in seconds. All .txt files represent 1.5 Ma injection episodes.
